# Bronchoscopic Valve Management of Persistent Air‐Leaks in Mechanically Ventilated Patients

**DOI:** 10.1111/1759-7714.70127

**Published:** 2025-07-17

**Authors:** Alfonso Fiorelli, Gaetana Messina, Francesca Capasso, Giuseppe Vicario, Davide Pica, Vincenzo Boniello, Massimo Ciaravola, Roberto Alfano, Fausto Ferraro

**Affiliations:** ^1^ Thoracic Surgery Unit University of Campania “Luigi Vanvitelli” Naples Italy; ^2^ Anesthesiology Unit, Benevento Hospital Naples Italy; ^3^ Anesthesiology Unit University of Campania “Luigi Vanvitelli” Naples Italy; ^4^ General Thoracic Surgery University of Campania “Luigi Vanvitelli” Naples Italy

**Keywords:** air‐leaks, bronchoscopy, endobronchial valve, ventilated patients

## Abstract

Management of persistent air‐leaks (PAL) in mechanically ventilated patients is challenging as most patients are refractory to standard treatments. Herein, we report the successful management of persistent air‐leaks in a case series of seven patients. Patients were mechanically ventilated and presented severe air‐leaks that persisted 17.5 ± 6.2 days after chest drainage insertion. Patients were deemed unfit for surgical resection while talc pleurodesis was unfeasible in five patients or performed without success in two. Valves treatment was performed at bedside in the Intensive Care Unit. Chest drainage was removed 5.5 ± 3.1 days after valve insertion, and all patients were weaned off the ventilator 8.8 ± 3.4 days later. All but one patient was discharged 26.8 ± 10.2 days following the procedure. One patient died at 35 days due to systemic sepsis not related to valves. Valves were readily removed in four patients at a mean of 149 ± 12.7 days after the implant. Our study confirmed the safe and effective use of EBV treatment for the management of PAL refractory to standard treatment in mechanically ventilated patients.

## Graphical abstract

Resolution of persistent air‐leaks after valve insertion.

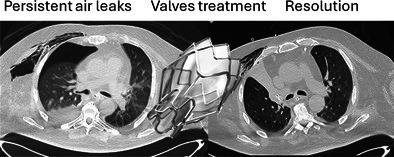

## 1 Background

Persistent air‐leaks (PAL) from alveolar pleural fistula (APF) in mechanically ventilated patients are a clinical condition associated with increased morbidity and mortality, and prolonged hospital stay. The management of such PALs is challenging as most patients are unfit for surgical resection due to severe comorbidities and are refractory to standard treatments [1, 2, 3, 4, 5, 6].

Herein, we report a case series of seven mechanically ventilated patients with PAL that were successfully treated by endobronchial one‐way valves (EBV, Zephyr EBV; Pulmonx Corporation, Redwood City, California, USA) insertion.

### 1.1 Study Population

The data were summarized in Table 1. The study population included seven consecutive patients (all male; mean age: 68.8 ± 7.7 years old) observed between January 2019 and March 2025. Patients were mechanically ventilated through tracheostomy (*n* = 5) or orotracheal tube (*n* = 2) and developed severe air‐leaks (grade 3 according to Macchiarini's scale [6]) from APF that persisted for 17.5 ± 6.2 days after chest drainage insertion. Talc pleurodesis was performed in two cases without success, while in five cases it was not feasible due to the lack of lung expansion. All patients were deemed unfit for surgical resection and thus scheduled for EBV treatment.

**TABLE 1:** Characteristics of study population.

Variable	Patient 1	Patient 2	Patient 3	Patient 4	Patient 5	Patient 6	Patient 7	Mean ± SD
Age	68	66	71	75	77	55	69	68.8 ± 7.7
Sex	Male	Male	Male	Male	Male	Male	Male	
Underlying disease	COPD, emphysema	COPD	COPD	COPD	COPD, Emphysema	COPD, emphysema	COPD, emphysema	
Ventilation	Tracheostomy	Tracheostomy	Tracheostomy	Trachestomy	Tube	Tracheostomy	Tube	
Air‐leak duration before valve implant (days)	18	20	25	25	10	15	10	17.5 ± 6.2
Air‐leak scores (Macchiarini's scale)	3	3	3	3	3	3	3	3
Treatment before valve implant	None	None	Talc pleurodesis	Talc pleurodesis	None	None	None	
No of segments treated	3	5	3	3	3	3	3	3.2 ± 0.7
Treated segments	R1; R2; R3	L1; L2; L3; L4; L5	R1; R2; R3	L1; L2; L3	R1; R2; R3	R1; R2; R3	R1; R2; R3	
No of valves implanted	3	2	3	1	3	4	3	2.7 ± 0.9
Valve size	4.0 EBV	5.5 EBV	4.0 EBV	5.5 EBV	4.0 EBV	4.0 EBV (2) 5.5 EBV (2)	4.0 EBV	
Procedure time (minutes)	35	15	29	10	27	45	37	28.2 ± 12.3
Lobe collapse on CT scan	Complete	Complete	Complete	Complete	Partial	Complete	Partial	
Treatment after valve implant	None	None	None	None	Talc pleurodesis	None	Talc pleurodesis	
Time to air‐leak resolution after valve implant (days)	1	1	1	1	5	1	7	2.4 ± 2.5
Time to chest drain removal after valve implant (days)	3	4	3	5	10	4	10	5.5 ± 3.1
Time to weaning off ventilator (days)	5	6	8	9	14	7	13	8.8 ± 3.4
Time to discharge from ICU (days)	43	24	18	Died	36	20	20	26.8 ± 10.2
Time to valve removal (days)	147	134	150	No	165	No	No	149 ± 12.7

The study was approved by the Local Ethics Committee of our hospital (number code: 833.18/18) and a signed informed consent form was obtained from the patients' family. The clinical data were available on request due to privacy.

### 1.2 Procedure

All procedures were performed at bedside in the Intensive Care Unit (ICU) under deep sedation and with administration of Cis‐Atracurium for muscle relaxation. The ventilator parameters were: low tidal volume around 5–6 mL/kg of predicted body weight, to minimize the risk of barotrauma and dynamic hyperinflation; respiratory rate 10–12 breaths per minute; an inspiratory‐to‐expiratory (I:E) ratio of approximately 1:2.5–1:3; moderate PEEP, 5–8 cm H_2_O, FiO2 65%–70%, pressure‐controlled ventilation mode. The flow was managed by AutoFlow setting to ensure that the set tidal volume was applied with the necessary minimum pressure for all mandatory breaths using a decelerating flow and allowing spontaneous breathing of the patient. AutoFlow delivered a decreasing flow to reach the set tidal volume and to avoid pressure peaks in volume‐controlled modes. If the resistance or compliance changes, the pressure adapted gradually to administer the set tidal volume. After the procedure, the ventilation mode was unchanged, and the FiO2 was reduced by 45%–50% as an improvement in oxygenation was obtained.

A flexible bronchoscope was inserted through the tracheal cannula or orotracheal tube. To identify the source of the air‐leaks, a Fogarty balloon catheter was sequentially inserted within the bronchial segments, and the air‐leak rate was assessed through the chest tube. In case of resolution and/or reduction of the air‐leaks, the valve was placed in a standard manner in the culprit segments.

The EBVs were deployed in the right upper lobe in five cases and in the left upper lobe in two cases. The total bronchial segments treated were 23, with a mean of 3.2 ± 0.7 segments for each patient. A total of 19 valves was used (mean of 2.7 ± 0.9 valves for each patient): five large valves (EBV 5.5) and 14 small valves (EBV 4.0). The mean procedure time was 28.2 ± 12.3 min. A recording of the procedure is provided in Video S1.

## 2 Results

**FIGURE 1:**
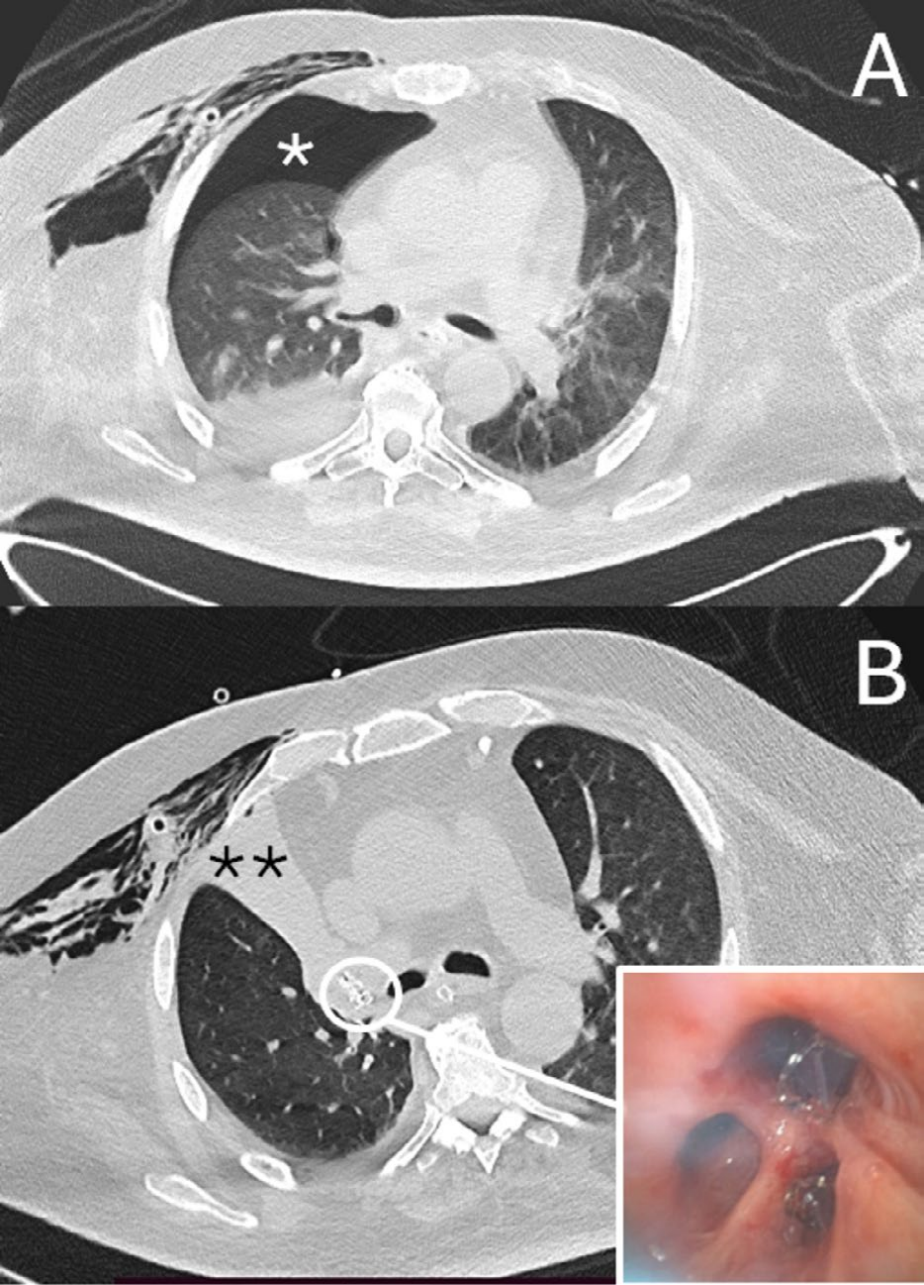
Persistent air‐leaks due to fistula of right upper lobe with pneumothorax (*). After valve insertion (insert), complete collapse of right upper lobe with fistula closure (**) was obtained, resulting in resolution of air‐leaks.

**FIGURE 2:**
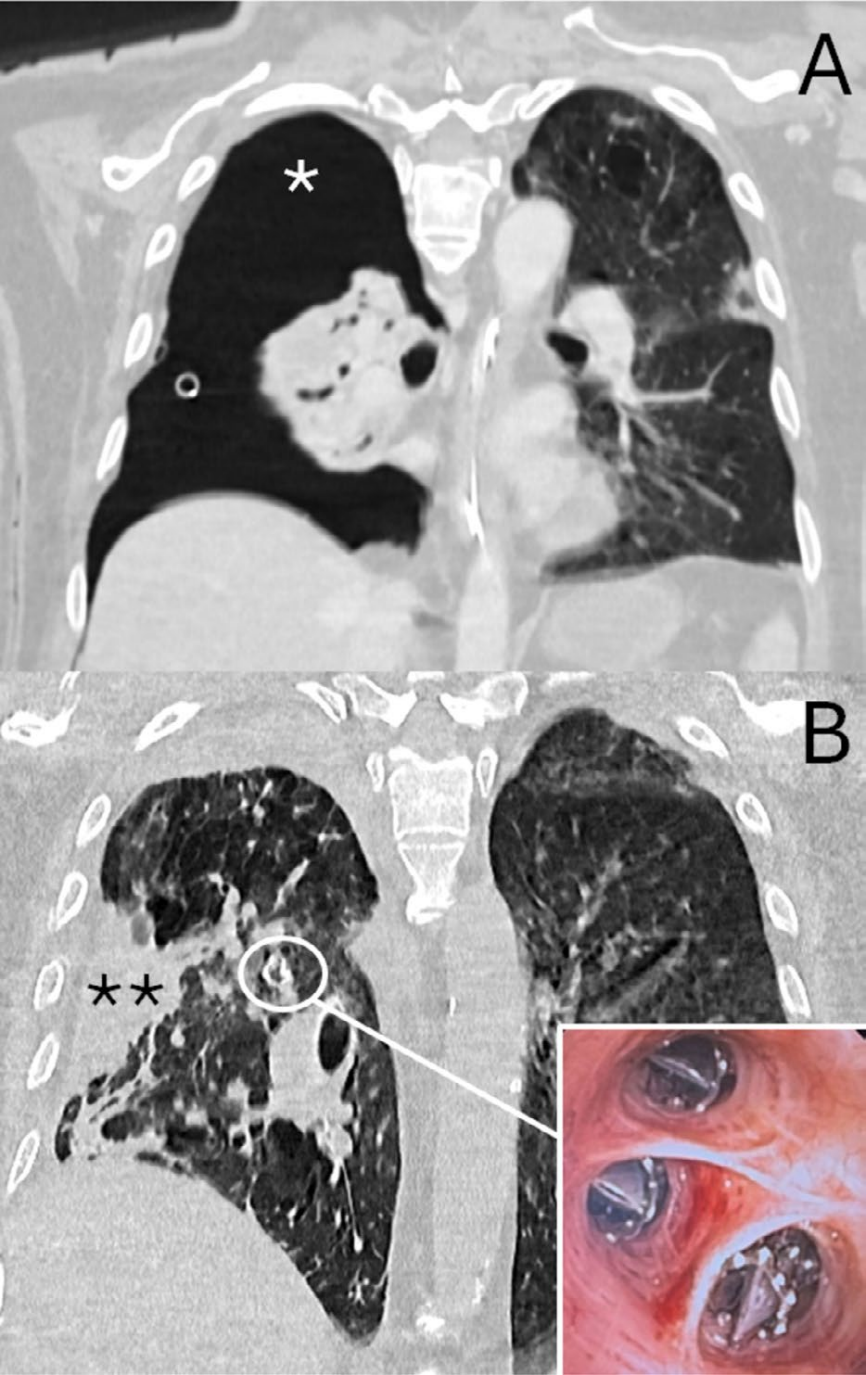
Large right pneumothorax (*) due to severe air‐leaks from fistula of right upper lobe. The insertion of valve (insert) allowed the fistula closure by the collapse of right upper lobe (**), resulting in lung expansion.

Complete (*n* = 5) and partial collapse (*n* = 2) of treated lobe were obtained after the procedure. No complications were observed before and after the procedure. Air‐leaks ceased within the first 24 h in five patients and were significantly reduced in the remaining two. In these cases, talc pleurodesis was then performed, obtaining the resolution of air‐leaks. Chest drainage was removed 5.5 ± 3.1 days after valve insertion, and all patients were weaned off from the ventilator 8.8 ± 3.4 days later. All but one patient were discharged from ICU at 26.8 ± 10.2 days following the procedure and transferred to the rehabilitation unit. One patient died at 35 days from systemic sepsis, which was not related to the valves. In four patients, valves were removed at 149 ± 12.7 days after the implant without problem. Clinical examples are shown in Figures 1 and 2. The clinical data were available on request due to privacy.

## 3 Discussion

PAL due to APF is a clinical condition due to abnormal communication between the pleural space and the pulmonary parenchyma that persists for more than 5 days, despite constant drainage of the thoracic cavity [1]. PALs are associated with increased morbidity and mortality and prolonged hospitalization. The American College of Chest Physicians (ACCP) guidelines [1] recommend surgical repair of APF in case of PAL, while non‐invasive treatments such as pleurodesis or endoscopic procedures are indicated in patients unfit for surgery [2, 3, 4, 5].

In our series, the barotrauma related to mechanical ventilation and the underlying lung disease were the main causes of APF. The PALs resulted in incomplete lung expansion and reduction of effective minute ventilation and oxygenation. The management remained challenging. The airway pressure due to mechanical ventilation maintained APF patent and prevented healing of injured parenchyma. Severe comorbidities prevented surgical management, while pleurodesis was not feasible in five patients as the lung did not remain inflated due to the presence of severe air‐leaks and failed in two. Thus, patients were considered for EBV treatment that has frequently been used to manage PAL after pulmonary resection in non‐critically ill patients [2, 3, 4, 5]. Conversely, information regarding EBV placement in mechanically ventilated patients with PAL is rare and limited to sporadic case reports. Lalla et al. [7] used EBV as salvage therapy in a mechanically ventilated patient with intractable life‐threatening haemoptysis. Following the atelectasis of the affected lobe, haemoptysis resolved and the patient was liberated from mechanical ventilation. Kalatoudis et al. [8] obtained the closure of bronchopleural fistula with EBV placement and liberation from mechanical ventilation in three patients who had acute respiratory distress syndrome (ARDS) with PAL. Similarly, Ghiani et al. [9] reported that EBV deployment facilitated weaning from extracorporeal membrane oxygenation (ECMO) in a patient with ARDS and PAL.

Our results confirmed the effectiveness and safety of EBV treatment for PAL in mechanically ventilated patients as previously reported in non‐critically ill patients. Complete resolution of air‐leaks was obtained in five cases. In the remaining two patients, EBV deployment significantly reduced the air‐leaks, allowed the complete expansion of the lung, and the resolution of air‐leaks after pleurodesis. For the success of the procedure, it was crucial to identify the culprit bronchial segments to treat by occluding with a balloon catheter. The integrity of the fissures was not routinely evaluated using StratX Lung Assessment as is done for emphysematous patients scheduled for bronchoscopic lung volume reduction with EBV [10]. Thus, the presence of a complete interlobar fissure could explain the lack of atelectasis of the treated lobe observed in two patients. No complications were registered during the insertion, neither high pressure of mechanical ventilation impair valve function and/or favor valve dislocation. Furthermore, the beneficial effect of lung volume reduction correlated to the atelectasis of the treated lobe improved oxygenation, resulting in a FiO2 reduction. If patients needed aspiration, we recommend performing this procedure by flexible bronchoscopy to avoid that the blind tip of the catheter aspiration could dislocate valves.

The less invasiveness and the reversibility were the main advantages of EBV treatment in these frail patients. The valves were inserted at ICU bedside without needing patient transfer to the operating room and could be easily removed if complications developed. In this series, the valves were removed in four out of seven patients after a mean duration of 149 ± 12.7 days after implant despite the current recommendation for removal after 6 weeks. In contrast to prior reports [2, 3, 4, 5], our patients were not discharged home after resolution of PAL, but to a rehabilitation unit to regain strength and function and to reduce the ICU stay. Thus, the valves were removed after patients had completed the rehabilitation program. The cost of the valves may be the main limitation of this strategy; however, it could be balanced by the reduction in the length of ICU stay.

In conclusion, our study confirmed the safety and effectiveness of EBV treatment for management of PAL refractory standard treatment in mechanically ventilated patients. Early EBV treatment in the course of a PAL could reduce the ICU stay, overcoming the valve costs. Our preliminary findings should be verified in larger and prospective studies.

## Author Contributions

A.F.: write and conceptualization. G.M., F.C., G.V., D.P., V.B., M.C., R.A.: data and figures collection. F.F.: supervision.

## Conflicts of Interest

The authors declare no conflicts of interest.

## Supporting information


**Video S1.** The video highlights the main steps of the valve treatment for management of persistent air‐leaks from fistula of right upper lobe.
